# Plains Medical Society

**Published:** 1891-08

**Authors:** 


### The Plains Medical Association.

In response to an informal call by letters addressed to the individual resident physicians of the Panhandle country, a large number of men belonging to the profession met at the office of Dr. J. W. Cartwright, in the city of Amarillo, on the 10th inst.

Dr. V. P. Kennedy, of Amarillo, was elected as temporary chairman and Dr. Johnson, of Washburn, as temporary secretary.

Dr. Cartwright, of Amarillo, then delivered an earnest welcome address, strongly urging the organization of a medical association in and for the country composed of the counties surrounding Amarillo, having for its object the social intercourse of its members and their scientific improvement.

An election for permanent officers was then held, resulting in the choice of Dr. Patton, of Claude, as President, and Dr. J. W. Cartwright as Vice-President. Dr. Johnson, of Washburn, was elected as Permanent Secretary, and Dr. T. F. McGee, of Amarillo, Treasurer.

A committee of five, consisting of Drs. McGee, Gore, Matthews, Kennedy and Cartwright were appointed to draft a constitution and by-laws, to report at next meeting.

The President appointed Drs. Gore and Matthews as essayists to prepare papers, to be read at the next meeting of the Association, upon subjects connected with medicine or surgery.

The society then adopted for its name "The Plains Medical Association." An adjournment was then made to meet in Amarillo on the 10th day of November, 1891.

